# Socioeconomic inequalities in access and use of skilled birth attendants during childbirth in Ghana: a decomposition analysis

**DOI:** 10.1186/s12884-021-04290-7

**Published:** 2021-12-31

**Authors:** Aseye Kpodotsi, Elizabeth Aku Baku, Jo Hunter Adams, Olufunke Alaba

**Affiliations:** 1 Ghana Health Service HQ (PPMED), PMB Ministries, Accra, Ghana; 2 P.O.Box 3430, Accra, Ghana; 3 Division of Health Economics, School of Public Health and Family Medicine, Faculty of Health Sciences, University of Cape Town, Observatory 7927, Cape Town, South Africa

**Keywords:** Inequity, Socioeconomic inequality, Skilled birth attendants, Maternal health care utilization, Childbirth, Child delivery, Ghana

## Abstract

**Background:**

Equitable access to skilled birth attendance during delivery is vital for reducing global maternal deaths to 70 deaths per 100, 000 to achieve the Sustainable Development Goals (SDGs) by 2030. Although several initiatives have been implemented to reduce maternal mortality in Ghana, inequalities in access to skilled birth attendance during delivery still exist among women of different socioeconomic groups. This study assesses the socioeconomic inequalities in access and use of skilled birth attendants during delivery in Ghana.

**Methods:**

Research was conducted through literature reviews and document reviews, and a secondary data analysis of the 2014 Ghana Demographic and Health Survey (GDHS), a nationally representative survey. A total of 1305 women aged 15–49 years, who had a live birth the year before to the survey in the presence of a skilled birth attendant were analysed using concentration indices and curves. The indices were further decomposed to identify the major socioeconomic factors contributing most to the inequalities.

**Results:**

The results found that access to skilled birth attendants was more among women from rich households showing a pro-rich utilization. The decomposition analysis revealed that household wealth index, educational level of both mother and husband/partner, area of residence and mother’s health insurance coverage were the major contributing factors to socioeconomic inequalities in accessing skilled birth attendants during child delivery among Ghanaian women.

**Conclusion:**

This study confirms that a mother’s socioeconomic status is vital to reducing maternal deaths. Therefore, it is worthy to focus attention on policy interventions to reduce the observed inequalities as revealed in the study.

## Background

*“Having a health worker (e.g., doctor, midwife or nurse) with accredited proficiency in midwifery skills present at childbirth especially in rural areas, supported by adequate equipment, supplies and drugs, regulations that permit them to carry out necessary procedures and transport for referral in case of emergency as required, is perhaps the most critical intervention for making motherhood safer.”* [1]**.**

Maternal health which remains a global priority initiated a number of international discussions on the need for safe motherhood leading to first Safe Motherhood Conference held in Nairobi, Kenya, organised by WHO (World Health Organization), UNFPA and The World Bank, in 1987 [2]. The Conference was to bring awareness on the increasing number of women dying during pregnancy and childbirth and also develop measures to prevent these avoidable situations [2].

Following this in 2000, the United Nations organised a Millennium Summit where heads of states adopted eight (8) international development goals knows as Millennium Development Goals to be achieved by 2015. Among them was goal 5 – “*to improve maternal health and its target indicators, 5A – to reduce maternal mortality ratio by three-quarters by the year 2015 and the proportion of births attended by skilled health personnel*” [3]. At the end of 2015, a progress report on the MDGs [4] was published admitting shortfalls in many areas, particularly in Sub-Sahara Africa. In the area of improving maternal health, in a span of 25 years, the number of skilled deliveries globally had increased to 71% from 59% in 1990 [5]. Despite the achievements in skilled birth assisted deliveries, the report revealed wide regional disparities especially in the Sub-Saharan region of Africa. Reports from the region in 2014, showed that there was only a 13% increase in skilled birth deliveries in a 25-year period. That is from 43% in 1990 to 59% in 2014 [5]. Investigations showed that severe differences in access to and use of skilled health personnel persisted within and across regions. Within regions and taking a look at geographical locations revealed difference between rural and urban areas where only 56% of births in rural areas were attended by skilled health personnel, compared to 87% in urban areas [6]. Given the uneven achievements and shortfalls from the MDGs, it was necessary to build new ambitions to achieve a better and more sustained future for all building on the successes of the MDGs. This opened doors to the Sustainable Development Goals (SDGs) intended to be achieved by the year 2030. The SDGs outlines 17 development goals and 169 targets in the areas of economic, social, and environmental [7]. The SDGs highlighted the importance of continued attention to maternal and new-born health through a more pragmatic approach. Considering this, a critical progress indicator was adopted for SDG 3 for reducing preventable maternal mortality by the year 2030 as the “proportion of births assisted by skilled health personnel” (SDG indicator 3.1.2) [8].

Access to skilled birth attendance (SBBA) is a key strategy to reduce the global maternal mortality ratio [5]. Therefore, this indicator serves as a proxy for use of maternal healthcare services. There is a strong correlation between skilled birth attendance and maternal mortality ratio. Studies have established evidence that high skilled birth attendance leads to low maternal mortality ratios [9–11]. Although there have been worldwide efforts to tackle the problem of increasing high maternal mortalities, estimates from most countries have proven slow and challenging due to lack of equitable access and use of maternal health services despite the existence of a well-functioning health care system. Several studies have also highlighted socioeconomic factors as a major influence for the low utilization of maternal health services [12–15].

Skilled birth attendance is vital in the pregnancy-childbirth continuum because most maternal deaths arise from childbirth complications [16]. Therefore, ensuring equity in access to skilled delivery services such as skilled birth attendance is essential to improving maternal health in Ghana. Over the years, there has been a lot of improvement towards reducing the high maternal mortality ratio due to preventable pregnancy-childbirth complications. Reports have shown that the majority of these deaths occur usually during childbirth and the immediate postpartum period (48 h after birth) [17].

High maternal mortality ratio^1^ is commonly reported in the least developed countries in the world. According to the 2015 MDG Report, a total of 295,000 maternal deaths in were due to preventable pregnancy and childbirth-related complications. Nearly 86% (254,000) of these deaths (295,000) occurred in sub-Saharan Africa and Southern Asia with Sub-Saharan Africa accounting for over 66% (196,000) and Southern Asia recording 20% (58,000) [18].

In Ghana, findings from the 2017 Ghana Maternal Health Survey (GMHS) reported the pregnancy-related mortality ratio (PRMR)^2^ as 343 deaths per 100,000 live births compared to the global rate of 210 deaths per 100,000 live births [19]. Further reports have suggested that direct causes account for majority (67%) of all maternal deaths with the most frequent causes being obstetric haemorrhage (30%) and hypertensive disorders (14%) [19].

Moreover, it has been confirmed that about 16 to 33% of these complications can be avoided by the assistance of a skilled birth attendant at childbirth [20]. Given this, Ghana initiated the Reproductive Health Strategic Plan to help improve maternal health. This was done by expanding women’s access to skilled attendance at delivery, increasing the availability of comprehensive essential obstetric care to treat pregnancy complications, and ensuring an effective referral and transport system to cater for pregnant women with complications to reduce pregnancy-related mortalities and morbidities [21].

According to the 2014 Ghana Demographic and Health Survey (GDHS) Report, Ghana has achieved a 74% coverage on skilled birth attendance [16]. Despite this high coverage, unequal access to skilled delivery services exists due to differences across geographic locations, socioeconomic groups, and socio-demographic factors. The GDHS 2014 Report recorded that, urban dwellers recorded 90% of births attended by skilled birth attendants as against 58% in rural areas. In addition, women from richer households recorded 96% compared to 49% from women living in poorer households [16]. Therefore differences within societies have become key determining factors of access and use of maternal health services during and after pregnancy [10–12, 16, 22].

Levesque et al. and Andersen in their conceptual framework of access and utilization of health services have studied the differences in health seeking behavioural patterns and grouped these variations into demand-side factors and supply-side factors [23, 24]. The demand-side factors include poor roads and long distances to health facilities, financial barriers, lack of employment opportunities, low educational attainment, low socioeconomic status, etc. while the supply-side factors include inadequate health facilities, staff attitude, lack of quality healthcare service, among others.

Studies have shown the existence of huge gaps in skilled birth attendance during delivery across different socioeconomic groups [25]. Some studies from low and middle-income countries have established a link between wealth, education, employment and skilled birth delivery [26–29]. For example, a study conducted in Namibia on decomposing inequities in skilled attendance at birth confirmed that wealth and education-related inequalities of the mother are the main determinants of inequities in access to skilled birth attendance [30].

It is in this regard that, the study seeks to examine the extent to which socioeconomic inequalities contribute to access to and use of skilled birth attendants and the underlying factors that contribute to these inequalities in the Ghanaian context.

## Methods

### Data source

The data used in this study was derived from the 2014 Ghana Demographic and Health Survey, which was collected by the Demographic and Health Surveys (DHS) Program. Information for the analysis was drawn from the DHS Women’s Questionnaire focusing on women who gave birth in the preceding the survey year. A two-stage sampling method was employed. A total of 427 clusters were selected in the first stage. Employing a systematic sampling for the second stage, 30 households were selected from each cluster totalling 12,831 households being selected of which 11,835 households were successfully interviewed. From the 11,835 households that were successfully interviewed, 9656 women aged 15–49 years were eligible for an individual interview at a response rate of 97% [16]. For this study, 1305 women between the ages of 15–49 years who had live births the year preceding the survey year were selected. The choice of a year before the survey date was to avoid the issue of recall bias from respondents.

### Study variable

#### Outcome variable

The outcome variable for this study is whether women who had a live birth the year preceding the interview year had deliveries assisted by skilled birth attendants or not. The outcome variable is a binary outcome where a value of “1” was given if the delivery was assisted by a skilled birth attendant and “0” if the delivery was not. A skilled birth attendant in this study was defined as a trained and licenced health professional i.e., a doctor, nurse/midwife or community health officer who provides basic and emergency healthcare services to women and their new-borns during pregnancy, delivery, and the immediate postpartum period (i.e., the first 48 h after delivery). Information on delivery assisted or attended by a skilled birth attendant was based on the question “*Who assisted in the delivery of (NAME OF CHILD*)” in the women’s questionnaire?

#### Predictor variables

##### Socioeconomic status (SES)/ factors

Socioeconomic factors are non-medical factors that influence health outcomes. They are social and economic experiences and realities that help mould one’s personality, attitude, and lifestyle. These factors can also define regions and neighborhoods and have an important influence on health inequities - the unfair and avoidable differences in health status seen within and between countries. In countries at all levels of income, health and illness follow a social gradient: the lower the socioeconomic position, the worse the health.

Research has shown that social determinants can be more important than health care or lifestyle choices in influencing health [31–33].

Economic status is measured by income. Social status is measured by education, and work status is measured by occupation. Each status is considered an indicator, although they are related, they do not overlap [34].

##### Household wealth index

The household wealth index is a measurement of the cumulative living standard of a household such as ownership of selected assets (e.g., televisions, radio, bicycles, etc.), sanitation facilities, types of water access, among others using the principal component analysis [31]. The household wealth index has been used in many demographics and health survey (DHS) reports to measure inequalities in household characteristics, access and use of health services, and health outcomes [32, 33]. The household wealth index is considered a more reliable measure than income and consumption because it represents a long-term standard of living of a household which allows for the identification of problems particular to the poor, such as unequal access and use of health services, etc. [34]. The household wealth index is calculated using a household’s ownership of selected items such as televisions and bicycles; materials used for housing construction; and types of water access and sanitation facilities [35]. A technique known as the principal component analysis was developed by Filmer and Pritchett to calculate the wealth index [36]. The household wealth index is usually divided into five wealth quintiles making the difference between the poor and rich very evident [31]. For this study, wealth was grouped into 5 quintiles – poorest (Q_1_), poorer (Q_2_), Middle (Q_3_), richer (Q_4_) and richest (Q_5_).

##### Education

Education is a process (occurring at home, school, family, and community) and a product (attained through formal and experiential learning), which is considered as one of the most widely used indicators of socioeconomic status [37]. Education is an attribute of a person and an essential factor of a person’s health.

There has been growing global recognition of the interdependency between education and health. The World Health Organization (WHO) posits that a person is unhealthy if he/she is unable to conduct him/herself effectively and achieve some level of ‘social well-being’. The Incheon Declaration states that quality education develops skills, values, and attitudes that enable an individual to lead a healthy and fulfilled life and make informed decisions [38].

There is good evidence that education is strongly linked to health outcomes and determinants of health such as healthy lifestyles and behaviours, health service utilization, etc. Therefore, people with higher educational levels may have better economic conditions which help them afford better and quality healthcare services, develop better information processing and abilities required to make better-informed decisions about their health [39]. One major reason educational level is used as a measure of socioeconomic status for an adult is the reduction in the likelihood of reverse causation as education is complete before health status declines [16]. Inequality in education opportunities is found not only regarding individuals and social classes but also in terms of regions and territorial regions such as urban and rural areas [40].

According to the 2014 GDHS, education is self-reported which collects the highest level of education attained by both women and their husbands/partners [16]. For this study, education will be regrouped into three (3): 1) no education, 2) primary, and 3) secondary +.

##### Occupation/employment

Occupation is used interchangeably with employment as a measure of socioeconomic status, embodies both income and education hence, its influence on health. Occupation/Employment reflects the educational attainment required to obtain the job and income levels that vary with different jobs and within ranks of occupations. This is used to measure the effect of socioeconomic status on health due to its role in positioning individuals within the social structure [41].

Employment has social, psychological, and financial benefits to improve one’s health. This implies that having a well-paying job provides an individual with the financial means to access nutritious foods, quality healthcare, safe housing, etc. all of which impacts health directly. The established correlation between employment and health is that having employment leads to income and eventually having the means to seek better healthcare thereby improving health status [42]. For example, with employment one can seek better health care services on time because they can afford it. In this study, occupation was measured using *maternal employment status* categorized into two groups: *employed or unemployed*.

##### Other socioeconomic variables

Mother’s autonomy is an important predictor variable [43]. A mother’s autonomy was defined in the GDHS as a mother’s ability to decide on their health. This was derived from the question: a *person who usually decides on a mother’s health care*. The response options were: (a) mother alone, (b) mother and husband/partner, (c) husband/partner alone and (d) other (i.e., any other person besides the aforementioned). However, for this study, the responses were limited to three (3): (1) mother alone, (2) mother and husband/partner, (3) husband/partner alone.

Other predictor variables of interest for this study include mother’s age at birth, mother’s marital status, sex of household head, region of residence, area of residence, mother’s health insurance ownership, mother’s educational level, husband/partner’s educational level, mother’s autonomy, household wealth index, and mother’s employment status. The selection of predictor variables in this study was based on existing literature that reported a significant association with different maternal health care services.

### Statistical analysis

#### Data analysis

The study analysed the data using STATA 14 statistical software. The Adept software version 6 was used to calculate the concentration indices and curves of the socioeconomic inequalities in access and use of skilled birth attendants during childbirth, the concentration of the problem in the selected population and the contribution of the socioeconomic factors to the observed inequality among the population.

### Measuring inequalities

The study estimated and measured inequality in the health outcome (access and use of skilled birth attendants during childbirth) using the concentration indices (CI) and concentration curves (CC). Before inequity can be measured, the following are essential:

- An indicator of the health outcome of interest (dependent variable) i.e., delivery by a skilled health professional.
- a stratifying factor capturing the socioeconomic status against which the distribution is to be assessed (household wealth index), and
- a measure of socioeconomic inequality to quantify the degree of inequity in the indicator variable of interest (dependent variable).

**Fig. 1:**
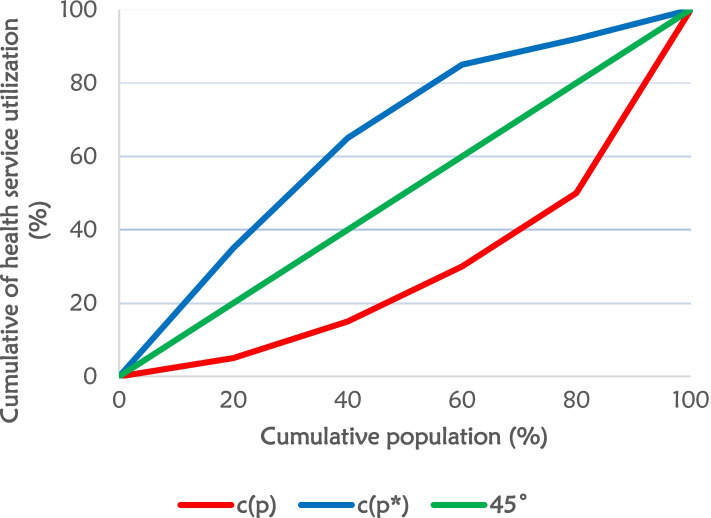
Concentration curve for health care utilization

The study used concentration curves and indices to measure socioeconomic status and inequalities that are essential in understanding the risk, burden, and impact of socioeconomic factors in accessing skilled birth attendants in Ghana. A concentration index (CI) is a relative measure (− 1 to + 1) of the extent to which a health outcome/variable is concentrated among the poor or the rich groups. The larger the absolute value of the concentration index (CI), the greater concentration of the inequality. A concentration curve (CC) plots the aggregate per centage share of health in a population against the aggregate per centage share of the population ranked according to their socioeconomic status (wealth) from the lowest to highest [44, 45]. As illustrated in Fig. 1, the concentration curves blue and red named C(p) and C(p*) respectively, are known as the line of inequality and may fall above or below the line of equality illustrated as green 45° line in the diagram. A concentration index is defined as twice the area between the line of equality (the green 45° line) and the concentration curve (either the blue C(p) or the red C(p*)). However, in a case where there is no income-related inequality, the concentration index is zero (0) meaning there will be no concentration curve above or below the line of equality as the concentration curve will be the same as the line of equality depicted in Fig. 1**.**

In this study, concentration indices (CI) were calculated to measure the magnitude of the inequality in the socioeconomic factors. The concentration index is defined as twice the area between the concentration curve and the line of equality (the 45-degrees line check the definition please). This is estimated as twice the covariance of the health care utilization and a person’s relative rank in terms of socioeconomic status, divided by the outcome mean [46]. This is presented in the formula below.

1 $$C=\frac{2}{\mu}\mathit{\operatorname{cov}}\left({h}_i,{r}_i\right)$$

Where C is the concentration index; *h*_*i*_ is the health variable index; *r*_*i*_ is the fractional rank of the individual *i* in the distribution of socioeconomic position; μ is the mean of the health variable and *cov* denotes the covariance.

The value of the CI measures the severity of socioeconomic inequality and vary between − 1 to + 1. A negative value implies that the health outcome is concentrated among those with lower socioeconomic status (i.e., the poor) showing a concentration curve above/to the left of the line of equality. A positive value indicates concentration among the higher socioeconomic status (i.e., the rich) showing a concentration curve below/to the right the line of equality. A CI value of zero implies no inequality. The larger the absolute value of CI, the greater the concentration of inequality [46, 47]. For example, if the health variable is ‘bad’ such as ill-health, a negative value of the concentration index means ill-health is higher among the poor, and vice versa.

### Decomposing the concentration index

Understanding and explaining the extent to which an underlying factor contributes to socioeconomic inequality has become of great interest to researchers and policymakers. The concentration index is commonly used to examine socioeconomic inequality in health [48, 49].

Decomposition estimations have mostly been used when the health outcome is a continuous variable (a numerical value that can be measured) using the Ordinary Least Square (OLS) regression model. However, given a situation where the dependent variable is binary like the use of skilled birth attendance during delivery or not as used in this study, the following need to be considered.

1. Regress the health outcome against its determinants using an appropriate model. This helps in finding the coefficients of the predictor variables (*β*_*k*_) as seen in eq. (2) below:

2 $$y=\alpha+{\textstyle\sum_k}\beta_kx_k+\varepsilon$$

Where *y* is the concentration index *(C)*, α is the y-intercept, β and χ are the predictor variable of health care demand and ɛ is the error term. Since most health outcomes are binary, a few studies have used different methods – Probit analysis [45] and logit analysis [49, 50]. Given the dichotomy nature of the dependent variable, the normalization process ensures that the CI is quantified in the range of-1 to 1 for any given health outcome [46]. Calculate the concentration indices of the health utilization outcome variable and the determinants using the equation below:

3 $$C={\textstyle\sum_k}\left(\beta_\kappa\frac{{\overline{\mathcal X}}_\kappa}\mu\right)C_k+\frac{GC_\varepsilon}\mu$$

Where *μ* is the mean of the outcome variable *y* in eq. 2 (i.e., mean of the deliveries by SBA) ${\overline{\mathcal{X}}}_k$ is the mean of ${\mathcal{X}}_k$, *C*_*κ*_ is the concentration index of determinant ${\mathcal{X}}_k$ x_k_ (defined analogously to *C)* and *GC*_*ε*_ is the generalised concentration index for the error term of (*ɛ)*. This equation shows that C is equal to the weighted sum of the concentration indices of the *κ* regressors, where the weight for ${\mathcal{X}}_{\kappa }$ is the elasticity of *y* for ${\mathcal{X}}_{\kappa}\left({\eta}_{\kappa }={\beta}_{\kappa}\frac{x_k}{\mu}\right)$. The residual component as captured by the last term reflects the income-related inequality in health that is not explained by systematic variation in the regressors, which should approach zero for a well-specified model.

### Ethical clearance

The GDHS 2014 sought ethical approval from the GHS Ethical Review Committee, Ghana and ICF Macro International Review Board, Maryland, USA. Further, written informed consent from each participant before enrolment was sought. For this study, ethical approval was received from the University of Cape Town Human Research Ethics Committee (HREC).

## Results

We included 1305 women in the analysis who had at least one birth in the past year preceding the survey. We considered women who accessed skilled birth attendants during childbirth in their last pregnancy.

### Sociodemographic characteristics of respondents

Out of 1305 women who had live births in the year before the study, 636 (49%) of the women who gave birth were between the ages of 25–34 years. More than half (60%) of the mothers were living in rural areas while 40% of the women live in urban areas. Eighty-eight per cent (88%) of the women were married. Regarding the household wealth index, 33% of the women were from the poorest quintiles while 11% were from the richest quintile. Forty-seven per cent (47%) of the mothers had secondary education while a third (33%) had no formal education. Considering the partner’s educational level, 52% had had secondary education or higher, however 27% had no education. A majority (71%) of the mothers were employed with only a few (29%) unemployed. Most (78%) of the households were headed by males and only 22% were headed by females. Approximately 77% of the respondents had health insurance coverage but 23% had none. For women autonomy, 605 (46%) of the mothers decided on healthcare together with their husbands/partners. This is presented in Table 1.

**Table 1:** Distribution of respondents by selected background characteristics

Characteristics	Number	Percent (%)
Mother’s age at birth		
15–24	376	28.8
25–34	636	48.7
35–49	293	22.5
Marital status		
Married	1145	87.7
Single	160	12.3
Area of residence		
Rural	786	60.2
Urban	519	39.8
Region of residence		
Ashanti	132	10.1
Brong Ahafo	123	9.4
Central	89	6.8
Eastern	104	8.0
Greater Accra	118	9.0
Northern	130	10.0
Upper East	144	11.0
Upper West	209	16.0
Volta	137	10.5
Western	119	9.1
Household wealth index		
Poorest (Q1)	434	33.3
Poorer (Q2)	274	21.0
Middle (Q3)	245	18.8
Richer (Q4)	200	15.3
Richest (Q5)	152	11.6
Mother’s educational level		
No education	436	33.4
Primary	261	20.0
Secondary+	608	46.6
Husband/partner’s educational level		
No Education	349	26.7
Primary	272	20.8
Secondary+	684	52.4
Mother’s employment status		
No	384	29.4
Yes	921	70.6
Sex of Household Head		
Female	291	22.3
Male	1014	77.7
Health Insurance Coverage		
No	301	23.1
Yes	1004	76.9
Mother’s autonomy		
Mother alone	233	17.9
Mother and husband/partner	605	46.4
Husband/partner	297	22.8

### Non-utilization of skilled birth attendants

From a total of 1305 women who had a live birth in the year before the interview, 28% of the deliveries were unassisted by skilled birth attendants. A breakdown by various socioeconomic stratifies is provided in Table 2.

**Table 2:** Non-utilization of skilled birth attendants during delivery by selected socioeconomic stratifies

Characteristics	Number (***N*** = 1305)			non-use of a skilled delivery
	No	Yes	Total Number	
	(***n*** = 366)	(***n*** = 939)		
**Mother’s age at birth**				
15–24	112	264	376	29.8
25–34	173	463	636	27.2
35–49	81	212	293	27.7
**Mother’s Marital Status**				
Married	320	825	1145	27.95
Single	46	114	160	28.75
**Area of residence**				
Rural	311	475	786	39.57
Urban	55	464	519	10.6
**Region of residence**				
Ashanti	35	97	132	26.52
Brong Ahafo	37	86	123	30.08
Central	5	84	89	5.62
Eastern	33	71	104	31.73
Greater Accra	43	75	118	36.44
Northern	13	117	130	10
Upper East	29	115	144	20.14
Upper West	130	79	209	62.2
Volta	12	125	137	8.76
Western	29	90	119	24.37
**Household wealth index**				
Poorest (Q1)	194	240	434	44.7
Poorer (Q2)	102	172	274	37.23
Middle (Q3)	60	185	245	24.49
Richer (Q4)	8	192	200	4
Richest (Q5)	2	150	152	1.32
**Mother’s educational level**				
No education	195	241	436	44.72
Primary	80	181	261	30.65
Secondary+	91	517	608	14.97
**Husband/partner’s educational level**				
No education	157	192	349	44.99
Primary	67	205	272	24.63
Secondary+	142	542	684	20.76
**Sex of Household head**				
Female	82	209	291	28.17
Male	284	730	1014	28
**Mother’s employment status**				
No	111	273	384	28.91
Yes	255	666	921	27.68
**Health insurance coverage**				
No	125	176	301	41.53
Yes	241	763	1004	24
**Mother’s autonomy**				
Mother alone	66	167	233	28.32
Mother and husband/partner	131	474	605	21.65
Husband/partner	119	178	297	40.07

The use of skilled birth attendants differed according to the various socioeconomic groups. Major differences were observed with area of residence, household wealth index, mother’s educational level, husband/partner’s educational level, health insurance cover and the region of residence. Regarding the area of residence as seen from Table 2 above, it is observed that the proportion of births unattended by skilled birth attendants were more in rural (39.6%) settlements compared to urban (10.6%) settlements. Among the household wealth index, mothers from the poorest households (45%) were more likely not to access skilled birth delivery compared to the richest households where only 1% of the mothers did not have skilled birth delivery. The lack of use of skilled birth attendants during childbirth was higher among the women with no educational level (44.7%) compared to their highly educated counterparts (15%) who had had a secondary level and higher education. Likewise, women whose husbands or partners were not educated (45%) were less likely to use skilled birth attendants. Based on the region of residence, the results showed the Upper west region as the region with the highest percentage (62.2%) of women not accessing skilled birth attendants during childbirth. This was followed by the Greater Accra region (36.4%), Eastern region (31.7%) and Brong Ahafo region (30.1%). Considering health insurance coverage, the study findings revealed that women who were not covered with health insurance did not use skilled birth attendants during childbirth (41.5%) compared to women who had insurance cover (24.0%). It was also observed that working mothers (29%) were more likely to use skilled birth attendants during delivery.

### Inequality associated skilled birth attendant

**Fig. 2:**
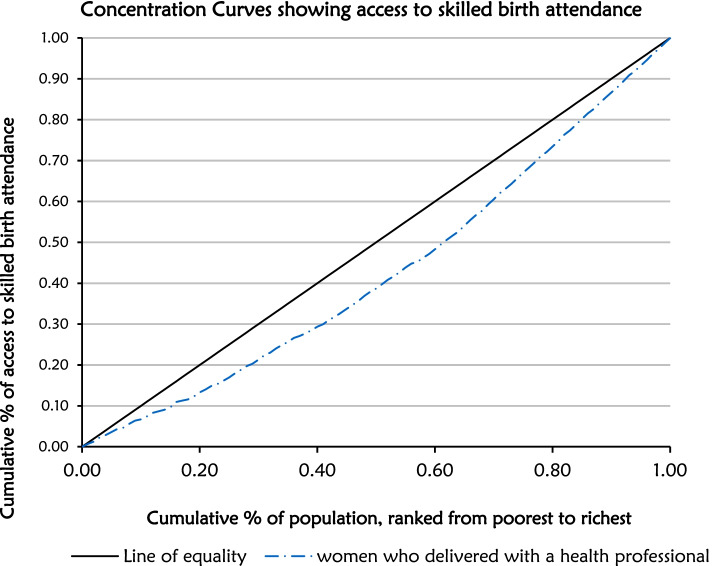
Concentration curve (CC) showing access to skilled birth attendants during delivery according to socioeconomic status

Figure 2 depicts the concentration curve of accessing skilled birth attendants during delivery according to socioeconomic status. The figure shows the existence of wealth-related inequality in accessing skilled birth attendants during delivery. The black diagonal line is the line of equality and the red curve below the black line represents the Concentration curve (CC). The farther the CC is below the line of equality the more concentrated the health outcome is among the rich. Therefore, the red concentration curve revealed that accessing skilled birth attendants during childbirth was concentrated among the rich. This implied that women from rich households were more likely to use skilled birth attendants compared to women from poor households. This is further confirmed by a positive concentration index of 0.188 suggesting a pro-rich inequality where the red curve lies below the diagonal line as shown in Fig. 2.

### Decomposition of underlying factors

Table 3 presents the results of the decomposition analysis explained the factors that contributed most to the observed inequalities accessing and using skilled birth attendants during childbirth. The contribution of each determinant depends on two factors; its impact on the delivery by a skilled birth attendant (elasticity) and how unequally distributed over wealth the determinant is (concentration index).

**Table 3:** Decomposing the socioeconomic inequalities in the utilization of skilled birth attendants during delivery in Ghana

Predictor Variables	Elasticities	CI	Contribution to CI	Contribution to CI
				**%**
**Mother’s age at birth**				
15–24	0	0	0	0.00%
25–34	0.0068	0.1045	0.0007	0.10%
35–49	0.0273	−0.1095	−0.003	−0.30%
**Mother’s marital status**				
Married	0	0	0	0.00%
Single	0	0	0	0.00%
**Area of residence**				
Rural	0.0671	0.4311	0.02893	2.90%
Urban	0	0	0	0.00%
**Region of residence**				
Ashanti	− 0.046	0.1219	− 0.0056	− 0.10%
Brong Ahafo	− 0.0641	0.0922	−0.0059	− 0.60%
Central	− 0.0484	0.5632	− 0.0273	−2.70%
Eastern	−0.0375	− 0.125	0.0047	0.50%
Greater Accra	−0.0544	−0.0105	0.0006	0.10%
Northern	−0.0692	0.3392	−0.0235	−2.30%
Upper East	−0.0305	−0.1418	0.0043	0.40%
Upper West	−0.1001	−0.6405	0.0641	6.40%
Volta	0	0	0	0.00%
Western	−0.0085	−0.5068	0.0043	0.40%
**Household wealth index**				
Poorest	0	0	0	0.00%
Poorer	0.0165	−0.3369	−0.0056	−0.60%
Middle	0.0304	0.0389	0.0012	0.10%
Richer	0.0794	0.4207	0.0334	3.30%
Richest	0.0595	0.811	0.0483	4.80%
**Mother’s educational level**				
No education	−0.0106	−0.4825	0.0051	0.50%
Primary	0	0	0	0.00%
Secondary+	0.0595	0.3013	0.0179	1.80%
**Husband/partner’s educational level**				
No education	−0.0219	−0.5563	0.0122	1.20%
Primary	0	0	0	0.00%
Secondary+	−0.0542	0.1732	−0.0094	− 0.90%
**Mother’s employment status**				
No	0.0205	−0.0115	−0.00024	0.00%
Yes	0	0	0	0.00%
**Sex of Household Head**				
Male	−0.006	0.1064	−0.00064	0.00%
Female	0	0	0	0.00%
**Health insurance cover**				
No	0.1009	0.0441	0.00445	0.40%
Yes	0	0	0	0.00%
**Mother’s autonomy**				
Mother alone	0.0032	0.0799	0.0003	0.00%
Mother and husband/partner	0.0243	0.0012	0	0.00%
Husband/partner	0	0	0	0.00%

Residual (unexplained) = 0.0004

The concentration index for childbirth in the presence of a skilled birth attendant showed that the estimated value of the relative contribution to the concentration index was negative in some socioeconomic factors such as woman’s employment status (− 0.01), mother’s educational level (no education = − 0.48), husband’s educational level (no education = − 0.56, primary = − 0.31), household wealth index (poorer = − 0.34) and the region of residence (Eastern = − 0.12, Greater Accra = − 0.01, Upper East = − 0.14, Upper West = − 0.64, Western = − 0.51). This, therefore, implies that individuals who were worse off in socioeconomic status were less likely to use skilled birth attendants during childbirth. The negative concentration indices indicate that the use of skilled birth attendants during childbirth was low and among the poor. In addition, the absolute values reveal a strong correlation between the mother’s educational level, husband/partner’s educational level and wealth and the use of skilled birth attendants during childbirth compared to the other socioeconomic factors.

A pro-rich utilization of skilled birth attendants during childbirth among Ghanaian women between the age of 15–49 years old was seen among women who had health insurance coverage; resided in urban areas; had had some form of education; whose husbands had had some form of education (secondary+); from middle and rich households; and who was in the Ashanti, Brong Ahafo, Central and Northern regions.

## Discussion

Using data from the 2014 GDHS, the study determined the factors that affect access and use of skilled birth attendants during childbirth among women aged 15–49 years who had at least one live birth the year preceding the year of the survey. Consequently, the study sought to quantify the factors’ contributions to socioeconomic inequalities using decomposition analysis.

The study result showed that a pro-rich concentration (0.188) indicating that more rich women accessed and used skilled birth attendants. The finding is consistent with the results of other studies where skilled birth attendants during childbirth was mostly accessed by women from rich households [30, 51, 52].

The study result showed unequal access and use of skilled birth attendants during childbirth regarding household wealth index, area of residence, region of residence, mother’s educational level, husband/partner’s educational level, and mother’s health insurance cover. The study findings revealed that the socioeconomic status of a mother plays a crucial role in the access and use of skilled birth attendants during childbirth.

Inequality in access and use of skilled birth attendants was found to be more predominant between the mothers in the poorest (4%) and richest (44.7%) groups within the wealth quintiles. This revealed a significant positive association for mothers in the richest quintiles as compared to women in the poorest quintiles. These findings supported previous studies that demonstrated unequal access and use of skilled birth attendants in childbirth [9, 10, 53–55]. This may be due to high financial burdens such as the cost of transportation, inpatient cost as well as delivery cost. Mothers from rich households were more likely to use skilled birth attendants during childbirth because they could afford to pay for the financial cost associated with its use as compared to mothers from poor households. For instance, in the case of caesarean section, the poor mother may find it difficult to pay for such a service so the family may feel hesitant to access the service even though they may be aware of its necessity.

The study also illustrated that area of residence was significant in influencing access and use of skilled birth attendants during childbirth in Ghana where urban dwellers are more likely to access and use skilled birth attendants. This is in line with studies conducted in Mali, Namibia, Sudan, Malawi, Sierra Leone, Niger, and India [22, 26, 29, 30, 51] which found that mothers living in urban areas were more likely to access and use skilled birth delivery in childbirth. This suggests that women living in urban areas generally have easier physical access to health facilities (i.e., availability of health facilities everywhere resulting in shorter distances to and from health facilities) and healthcare services and staff (availability of comprehensive health services and proficient health staff) compared to rural dwellers.

Moreover, the study analysis showed that the region of residence was also significantly associated with women’s access and use of skilled birth attendants in Ghana. The Upper West region had the highest percentage (62.2%) of women not using skilled birth attendants during childbirth compared to the Central Region with the least percentage (5.6%) of women not using skilled birth attendants. Other regions that showed a high percentage of women not using skilled birth attendants were Greater Accra Region (36.4%), Eastern Region (31.7%), Brong Ahafo Region (30.1%), Ashanti Region (26.5%), Western Region (24.4%) and Upper East Region (20.1%). The remaining regions, Northern Region, Volta Region, and Central Region had percentages less than 10%, that is, 10, 8.8, and 5.6%, respectively. This finding was supported by a study conducted in Uganda which showed that region of residence influenced access and use of skilled birth attendants [53].

Additionally, the mother’s educational level to access and use of skilled birth attendants was a significant influence on access and use of skilled birth attendants. According to the study analysis, mothers with no education (44.72%) were less likely to use skilled birth attendants than mothers with secondary and higher education (14.97%). Other studies also confirm similar results [10, 15, 26, 49, 55, 56] where mothers with high educational levels are more likely to access and use skilled birth attendants. A High educational level suggests a high literacy level thereby the ability to identify health issues (e.g., identify danger signs related to pregnancy and childbirth), seek appropriate healthcare, and adopt healthy lifestyles and choices throughout their lifetime.

Similarly, husbands/partners who had higher educational levels (secondary and higher) were more likely to access and use skilled birth attendants during childbirth than husbands/partners with no/lower educational levels. A husband/partner with a higher educational level is likely to have higher knowledge and awareness of pregnancy complications [57].

Furthermore, the study findings showed a positive impact of health insurance coverage on access and use of skilled birth attendants during childbirth. Health insurance coverage (41.53%) increased a woman’s chance of accessing and using skilled birth attendants during childbirth compared to women without health insurance (24%). This is consistent with previous studies where health insurance ownership increased a mother’s access and use of skilled birth attendants [29, 58]. This infers that having ownership of health insurance gives a mother financial protection, all other things being equal, to afford healthcare services including access and use of skilled birth attendants during childbirth since they do not have to pay directly for health services.

## Conclusion

The decomposition analysis revealed that household wealth index, area of residence, region of residence, mother’s educational level and husband/partner’s educational level contributed significantly to the inequalities to access and use of skilled birth attendants during childbirth. The educational level of both mother and husband/partner poses major barriers to access and use of skilled birth attendants during childbirth. Therefore, scaling-up of health education by community-based organizations such as queen mothers’ associations, women groups and church groups may have beneficial effects on pregnant mothers accessing and using skilled birth attendants during delivery and hence prevent maternal mortality during delivery. The Ministry of Health together with the Ghana Health Service need to develop strategies that generate demand for skilled delivery services and reduce financial and geographical barriers to those services.

## Data Availability

Data can be accessed on the Demographic and Health Surveys program website (www.dhsprogram.org).

## Abbreviations

CC: Concentration Curve
CI: Concentration Index
CHPS: Community-based Health Planning and Services
DHS: Demographic and Health Survey
GDHS: Ghana Demographic and Health Survey
GHS: Ghana Health Service
HIV/AIDS: Human Immunodeficiency Virus/ Acquired Immune Deficiency Syndrome
HREC: Human Research Ethics Committee
MDGs: Millennium Development Goals
PRMR: Pregnancy-Related Mortality Ratio
RHSP: Reproductive Health Strategic Plan
SBA(s): Skilled Birth Attendance
SDGs: Sustainable Development Goals
SES: Socioeconomic Status
STI: Sexually Transmitted Infections
UNDP: United Nations Development Programme
WHO: World Health Organization

## Footnotes

1 For categorization, MMR is considered to be low if it is less than 100, moderate if it is 100–299, high if it is 300–499, very high if it is 500–999 and extremely high if it is greater than or equal to 1000 maternal deaths per 100,000 live births.

2 The pregnancy-related mortality ratio (PRMR) is defined as the number of pregnancy-related deaths per 100,000 live births. It is a ratio, rather than a rate because the denominator contains only live births and not all pregnant women who are at risk of maternal death.
